# Characterizing the drivers of seedling leaf gas exchange responses to warming and altered precipitation: indirect and direct effects

**DOI:** 10.1093/aobpla/plw066

**Published:** 2016-10-26

**Authors:** Nicholas G. Smith, Grace Pold, Carol Goranson, Jeffrey S. Dukes

**Affiliations:** 1 Department of Forestry and Natural Resources, Purdue University, West Lafayette, IN, USA; 2 Department of Biological Sciences, Purdue University, West Lafayette, IN, USA; 3 Purdue Climate Change Research Center, Purdue University, West Lafayette, IN, USA; 4 Department of Microbiology, University of Massachusetts, Amherst, MA, USA; 5 Department of Biology, University of Massachusetts, Boston, MA, USA

**Keywords:** Boston-Area Climate Experiment (BACE), climate change, photosynthesis, relative extractable water, respiration, soil moisture, stomatal conductance, *V*_cmax_

## Abstract

Anthropogenic forces are projected to lead to warmer temperatures and altered precipitation patterns globally. The impact of these climatic changes on the uptake of carbon by the land surface will, in part, determine the rate and magnitude of these changes. However, there is a great deal of uncertainty in how terrestrial ecosystems will respond to climate in the future. Here, we used a fully factorial warming (four levels) by precipitation (three levels) manipulation experiment in an old-field ecosystem in the northeastern USA to examine the impact of climatic changes on leaf carbon exchange in five species of deciduous tree seedlings. We found that photosynthesis generally increased in response to increasing precipitation and decreased in response to warming. Respiration was less sensitive to the treatments. The net result was greater leaf carbon uptake in wetter and cooler conditions across all species. Structural equation modelling revealed the primary pathway through which climate impacted leaf carbon exchange. Net photosynthesis increased with increasing stomatal conductance and photosynthetic enzyme capacity (*V*_cmax_), and decreased with increasing respiration of leaves. Soil moisture and leaf temperature at the time of measurement most heavily influenced these primary drivers of net photosynthesis. Leaf respiration increased with increasing soil moisture, leaf temperature, and photosynthetic supply of substrates. Counter to the soil moisture response, respiration decreased with increasing precipitation amount, indicating that the response to short- (i.e. soil moisture) versus long-term (i.e. precipitation amount) water stress differed, possibly as a result of changes in the relative amounts of growth and maintenance demand for respiration over time. These data (>500 paired measurements of light and dark leaf gas exchange), now publicly available, detail the pathways by which climate can impact leaf gas exchange and could be useful for testing assumptions in land surface models.

## Abstract (precis)

Climate change is expected to bring warmer temperatures and more variable precipitation patterns worldwide, patterns that will depend on the ability of the world's flora to take up carbon under these new conditions. We subjected deciduous tree seedlings growing in an old-field ecosystem in Massachusetts, USA to warming and altered precipitation. We found that leaf carbon uptake was greatest under the coolest, wettest conditions, an effect driven by increased soil water availability in these plots. Our findings suggest that warming may reduce leaf carbon uptake by decreasing soil moisture, an effect that will be exacerbated during drought periods.

## Introduction

Globally, terrestrial carbon exchange represents the largest flux of carbon between the Earth’s surface and the atmosphere (Le Quéré *et al*. 2012; IPCC, 2013) and studies have shown that land-atmosphere carbon cycle feedbacks are a major source of uncertainty in the Earth System Models used to project climate change (Friedlingstein *et al*. 2013). The flux of carbon between the atmosphere and land surface is dominated by photosynthetic carbon uptake by vegetation, as well as carbon release from vegetation and soils through respiration. Photosynthesis and plant respiration are variable and strongly influenced by climatic conditions (Wu *et al*. 2011; Lu *et al*. 2012), but scientific understanding of how these fluxes will be altered by climate change remains limited (Arneth *et al*. 2010). This is, in part, because the responses of these fluxes to experimental conditions are complex, as they are influenced by the scales considered as well as interactions among driving variables (Smith *et al*. 2014).

Climate change is expected to result in warmer temperatures and changes in precipitation patterns for most of the world (IPCC, 2013), including the northeastern USA (Hayhoe *et al*. 2007). Warming directly influences plant gas exchange. Short-term (seconds to minutes) warming typically increases enzymatic rates and, subsequently, rates of photosynthesis and respiration up to a peak, beyond which rates decline. This optimum occurs at a lower temperature in photosynthesis than respiration. In response to longer-term warming, photosynthetic and respiratory enzyme activity may show an acclimation response, resulting in rates that differ from those expected from short-term responses alone. The acclimated rates may be higher, lower, or similar to those observed without warming (Atkin *et al*. 2005; Way and Yamori 2014; Yamori *et al*. 2014). Warming may also influence photosynthetic rates by increasing leaf vapour pressure deficit, which reduces stomatal conductance and, subsequently, photosynthesis (Ocheltree *et al*. 2014). Warming may also reduce net photosynthesis (i.e. photosynthesis minus leaf respiration) if it increases leaf respiration to a greater degree than photosynthesis.

A meta-analysis of 24 experimental warming studies found that warming tends to enhance ecosystem photosynthesis (Wu *et al*. 2011), an effect which was shown to occur in 68 % of leaf-level studies (Way and Yamori 2014). Meta-analyses of respiration are less conclusive, with ecosystem-level studies showing an increase in aboveground respiration (Wu *et al*. 2011), and leaf-level studies indicating a decrease in respiration with increasing growth temperature (Slot and Kitajima 2014). These differences likely arise because of time and spatial scale incongruences, which are difficult to account for within and when comparing across meta-analyses.

Both warming and altered precipitation can affect soil moisture and, consequently, soil water availability to plants; while precipitation affects soil moisture directly by adding water to the system, warming effects are indirect and occur as the result of changes in evapotranspiration of water from plants and soil (Harte *et al*. 1995; Seneviratne *et al*. 2010). Soil water availability has been shown to influence leaf gas exchange (e.g. Camberlin *et al*. 2007; Ignace *et al*. 2007; Li *et al*. 2007; Llorens *et al*. 2004). Increases in soil moisture can increase photosynthetic carbon uptake as stomata open further, allowing CO_2_ to diffuse into leaves more quickly (Hsiao 1973; Chaves *et al*. 2009; Pinheiro and Chaves 2011). Conversely, stomata close in drier soils, slowing water loss and leading to a decrease in intercellular CO_2_ and, thus, photosynthetic rates (Potter *et al*. 1993; Shaw *et al*. 2002; Wu *et al*. 2011; Chaves *et al*. 2009; Pinheiro and Chaves 2011).

Respiration responses to soil moisture, and drought in particular, have not been consistent across studies (Pinheiro and Chaves 2011); some studies show that drought may inhibit respiration, similar to its effects on photosynthesis (Ribas-Carbo *et al*. 2005; Galmes *et al*. 2007; Gimeno *et al*. 2010; Flexas *et al*. 2006), but drought may also increase respiration as result of increased respiratory demand for ATP (Atkin and Macherel 2009) or increased maintenance respiration (Gratani *et al*. 2007; Slot *et al*. 2008). Alternatively, other studies have found no effect of soil moisture changes on respiration (Galmes *et al*. 2007; Gimeno *et al*. 2010). The response to water availability likely depends in part on photosynthetic supply of substrate (Gifford 2003; Van Oijen *et al*. 2010; Pinheiro and Chaves 2011), but the coupling of photosynthesis and respiration under different soil water conditions has not been well studied, particularly in natural areas and in the context of climate change.

Although soil moisture responses of leaf carbon exchange are well studied in potted plants, field studies are lacking, particularly in areas outside the Mediterranean region (Chaves *et al*. 2002, 2009; Pinheiro and Chaves 2011). As a consequence, model representation of soil moisture responses of leaf carbon exchange are still rudimentary, relying on simple scaling factors to adjust photosynthetic capacity (e.g. Oleson *et al*. 2010) or the relationship between photosynthesis and conductance (e.g. Zaehle *et al*. 2010) in response to moisture availability (Egea *et al*. 2011; Smith *et al*. 2014). Although simple, these formulations and the way in which they are implemented differ greatly between models (De Kauwe *et al*. 2013) and the different implementations have shown varying capacities to reproduce observed data (Keenan *et al*. 2010; Egea *et al*. 2011), indicating a need for more data describing these responses in the field (Smith *et al*. 2014).

In this study, we examined leaf-level gas exchange and growth in five species of tree seedlings in response to four levels of warming and three levels of precipitation across a single growing season. The species used were *Betula lenta*, *Betula populifolia*, *Prunus serotina*, *Quercus rubra* and *Ulmus americana*. These species vary in their current and projected ranges, with both *Betula* species being restricted longitudinally (suggesting precipitation sensitivity), and *B. populifolia* being restricted latitudinally (suggesting temperature sensitivity) relative to *P. serotina*, *Q. rubra* and *U. americana*, which have large current and projected ranges that span much of the eastern and midwestern USA (Prasad *et al*. 2007-ongoing; Iverson *et al*. 2008).

We hypothesized that warming would not directly influence observed rates of photosynthesis and respiration because of acclimation responses. We further hypothesized that both warming and precipitation change would influence soil moisture and that soil moisture would correlate positively with stomatal conductance and photosynthesis. We expected that the influence of soil moisture on respiration would mirror that of photosynthesis because of an increase in substrate supply for respiration. We expected that the soil moisture effect would be the result of a combination of stomatal and biochemical effects. As such, we expected warming to exacerbate the negative effect of reduced precipitation and to counteract the positive effect of added precipitation on leaf photosynthesis and respiration. In general, we expected the *Betula* species to be most sensitive to the climate treatments because of their smaller range sizes.

## Methods

### Research site Boston-Area Climate Experiment

All research was conducted at the Boston-Area Climate Experiment (BACE; Rodgers *et al*. 2012; Suseela *et al*. 2012), which is located in an old-field ecosystem at the University of Massachusetts’ Suburban Experiment Station in Waltham, Massachusetts, USA (42° 23′ 3″ N, 71° 12′ 52″ W). The site had a mean annual temperature of 9.3 °C and mean annual precipitation of 1180 mm, with similar amounts of precipitation falling in each month (NOAA National Climatic Data Center Cooperative Station ID 190535, January 1960–April 2009). The experimental area had a loam topsoil (0–0.3 m) over a gravelly sandy loam subsoil. The experiment had three blocks, each containing 12, 2 × 2 m plots (36 plots in total). Clear plastic ‘rainout’ shelters provided 50 % cover over the four ‘dry’ plots in each block, redirecting 50 % of the ambient rainfall to storage tanks. That captured rainwater was immediately distributed to an area encompassing the four ‘wet’ plots, creating three precipitation treatments: dry (50 % ambient), wet (150 % ambient), and ambient. Warming treatments were applied within the precipitation treatments. Ceramic heaters of different wattages were mounted 1 m above all corners of each plot. These supplied either no, low (200 W/heater), medium (600 W/heater) or high (1000 W/heater) levels of heating to each plot. Fake heater boxes were used to replicate non-warming effects of heaters (e.g. shading) in the unwarmed treatment. Infrared radiometres (Apogee Instruments, Logan, UT, USA) sensed the canopy temperature in the unwarmed and ‘high’ plots and a feedback control system (LabVIEW; National Instruments Corp, Austin, TX, USA) was set to maintain a 4 °C difference between the two. All heaters within a group of four plots were wired to the same circuit. The three precipitation treatments and four temperature treatments provided twelve climate treatments. All treatments were turned on by July 2008. The mean canopy temperature difference between the high warming and unwarmed plots across the 2011 growing season was 2.81 °C, with 0.27 °C greater warming achieved during the night than during the day, when greater convective heat losses often prevented the experimental infrastructure from achieving warming targets. The greater warming at night occurred on 61 % of the days across the measurement period. There was minimal variation in canopy temperature among precipitation treatments (±0.14 °C).

### Plot setup and species composition

The BACE was constructed in an old-field ecosystem. Each plot contained a mixture of common, mostly non-native grass and forb species (Hoeppner and Dukes 2012). In addition, seedlings of eight native tree species (*Acer rubrum, B.* *lenta, B.* *populifolia, Pinus strobus, Populus grandifolia, P.* *serotina, Q.* *rubra* and *U.* *americana*) were planted in four subplots within the main plots in late April 2011. Four seedlings of each species were planted in each 2 × 2 m plot (one individual per species per 0.5 × 0.5 m subplot). Due to high mortality of the other species, responses were analyzed for only a subset of species (*B. lenta*, *B. populifolia*, *P. serotina*, *Q. rubra* and *U. americana*). All species’ ranges extended northward beyond 45°N. *B. populifolia* is typically not found further south than 40°N, whereas the other three species’ ranges extend close to or further south than 30°N. *P. serotina*, *Q. rubra* and *U. americana* have longitudinal ranges that extend from the East coast of the USA westward beyond 90°W. The two *Betula* species have a similar eastern edge, but typically do not extend westward beyond 80°W for *B. populifolia* or 85°W for *B. lenta* (Prasad *et al*. 2007-ongoing). The geographical differences correspond well with niche space differences for these species, which indicate that the *Betula* species are not able to tolerate the low levels of rainfall (below ∼500 mm/year) that *P. serotina*, *Q. rubra* and *U. americana* can tolerate. In addition, the upper end of mean annual temperature tolerance is lower for *B. populifolia* (∼10 °C) than the other species evaluated (Prasad *et al*. 2007-ongoing). Future projections suggest an increase in abundance of *P. serotina*, *Q. rubra* and *U. americana* in the northeastern USA due to northerly range shifts, but a decrease in abundance of both *Betula* species, particularly *B. populifolia* (Prasad *et al*. 2007-ongoing; Iverson *et al*. 2008).

### Soil moisture measurements

The average relative extractable water (*θ*_R_) was monitored weekly at the site. *θ*_R_ is an estimate of the ratio of total extractable water (*θ*_T_) to maximum extractable water (*θ*_T,max_) available for uptake by plants across multiple soil layers throughout the rooting zone. *θ*_T_ was calculated using a function described by Vicca *et al*. (2012):

(1) $$\theta_{T}=(\theta_{S1}-\theta_{S1,\text{wp}})H_{1}+(\theta_{S2}-\theta_{S2,\text{wp}})H_{2}+\ldots (\theta_{\text{Sn}}-\theta_{\text{Sn},\text{wp}})H_{n}$$

where *θ*_Sn_ is the soil water content of a given layer n, *θ_Sn,wp_* is the soil water content at the wilting point for layer *n*, and *H*_n_ is the thickness of layer *n*. Here we measured *θ*_S_ at three layers (0–0.3, 0.45 and 0.60 m) using time domain reflectometry (100; Campbell Scientific, Logan, UT, USA) and permanently installed waveguides. *θ*_S_ across the 0-0.3 m range was estimated using vertical waveguides, while horizontal waveguides at 0.45 m depth were used to estimate *θ*_S_ across the 0.3–0.525 and 0.525–0.75 m range, respectively. An estimate of 0.14 m^3^/m^3^ volumetric water content (VWC) was used for *θ*_S,wp_ at 0–0.3 m depth, and 0.08 m^3^/m^3^ VWC was used for depths below 0.3 m (Saxton and Rawls, 2006). For *θ*_T,max_, estimated field capacity values of 0.28 m^3^/m^3^ VWC for 0–0.3 m depth and 0.18 m^3^/m^3^ VWC for depths below 0.3 m were used in place of *θ*_S_. In some cases, *θ*_S_ at a given depth could not be estimated due to equipment error. In those cases (9.5 % of data), we gap-filled the data using the linear relationship between values at that depth and the depth directly above it, which were strongly correlated in all cases. In cases where *θ*_R_ was estimated below zero or above one, estimates were set to zero and one, respectively.

***Gas exchange measurements.*** Gas exchange analyses were performed during three separate measurement periods within the middle of the growing season of 2011: late spring (5–8 June, day of year (DOY) 156–159), early summer (29 June–2 July, DOY 180–183), and midsummer (28–31 July, DOY 209–212). Two individuals per species were randomly chosen in each main plot; however, for some species during some measurement periods, one or no individuals of a species were measured in a given plot due to seedling death or lack of suitable leaves. Snapshot measurements of leaf carbon and water exchange, including net photosynthesis (*A*_n_), transpiration (*E*) and stomatal conductance (*g*_s_), were taken during midday hours (between 1000 and 1600 h) on the youngest fully expanded leaf of each seedling using a LI-6400 portable photosynthesis system (LI-COR Inc., Lincoln, NE, USA). Light within the chamber was set to a saturating level (1500 µmol m^−^^2^ s^−^^1^ PAR) provided by red/blue LED lights within the chamber of the LI-6400. Cuvette CO_2_ concentrations were set to 360 µmol mol^−^^1^. Leaf temperatures inside the cuvette were set to the leaf temperature read inside the cuvette by the internal thermocouple immediately following clamping on to the leaf and allowed to stabilize before readings were taken. Leaf vapour pressure deficit (*D*_leaf_) was allowed to stabilize, but not held constant.

Dark respiration (*R*_d_) measurements were taken the night following the photosynthesis measurements on the same leaf as the previous days’ photosynthesis measurements. Measurements were taken at least two hours after sunset with similar cuvette CO_2_ and airflow settings as the photosynthesis measurements. Temperatures inside the cuvette were set to the temperature read by the internal thermocouple following closure of the cuvette.

***Data analysis and statistics*.** We estimated daytime respiration (*R*_l_) by standardizing *R*_d_ values to rates at the leaf temperature observed for *A*_n_ using a variation of a temperature-dependent temperature sensitivity formula described by Tjoelker *et al*. (2001) such that:

(2) $$R_{l}=R_{d}{\left(3.22-0.046T_{d}\right)}^{\frac{T_{l}-T_{d}}{10}}$$

where *R* is the standardized rate, *T*_l_ is the leaf temperature at the time of the photosynthesis measurement (i.e. temperature in light), and *T*_d_ is the leaf temperature at the time of the respiration measurement (i.e. temperature in dark). From this, gross photosynthesis (*A*_g_) was calculated by adding *R*_l_ to *A*_n_ (e.g. *A*_g_ *=* *A*_n_ *+* *R*_l_). Then, the ratio of carbon lost to respiration to carbon gain through photosynthesis (*R/A*) was calculated as *R*_l_ divided by *A*_g_. We did not consider light inhibition of dark respiration, given its variability under differing environmental conditions (e.g. Kroner and Way, 2016) and the relative insensitivity of *A*_g_ to this effect.

To explore the role of biochemical and stomatal effects on our photosynthesis results, we calculated maximum rate of Rubicsco carboxylation (*V*_cmax_) using the one-point method (De Kauwe *et al*. 2015). This method operates under the assumption that our measured photosynthetic rates were carboxylation, rather than electron transport or phosphate utilization limited, at 1500 µmol m^−^^2^ s^−^^1^ PAR. We used the Farquhar *et al*. (1980) model to calculate *V*_cmax_ such that:

(3) $$V_{\text{cmax}}=\frac{A_{g}\left(C_{i}+K_{m}\right)}{C_{i}-\Gamma^{*}}$$

where *C_i_* is in the intracellular CO_2_ concentration as measured by the LI-6400, *K*_m_ is the Michaelis-Menten constant given by:

(4) $$K_{m}=K_{c}\left(1+\frac{o_{i}}{K_{o}}\right)$$

Γ*, *K*_c_ and *K*_o_ were estimated using leaf temperature using the equation

(5) $$f\left(T_{k}\right)=a^{*}\;\text{exp}\;\left(\frac{b\left(T_{k}-298.15\right)}{298.15T_{k}}\right)$$

where *T*_k_ is the leaf temperature in Kelvin, *R* is the gas constant (8.314 J mol^−^^1^ K^−^^1^), and parameters *a* (the rate at 25 °C) and *b* (J mol^−^^1^) describe the shape of the curve and are taken from Bernacchi *et al*. (2001). We then estimated the degree of stomatal limitation by calculating a modified *C*_i_ (*C*_i,mod_) that assumes no stomatal limitation as:

(6) $$C_{i,\text{mod}}=C_{a}$$

where *C_a_* is the external CO_2_ level (i.e., 360 µmol mol^−^^1^) (Long and Bernacchi 2003). Finally, the degree of stomatal limitation (*l*) was estimated as:

(7) $$l=1-\frac{A_{n}}{A_{n,\text{mod}}}$$

where *A*_n,mod_ is the rate of net photosynthesis (*A*_n,mod_) calculated using *C*_i,mod_ and equation (3) (Farquhar and Sharkey 1982).

We calculated rates of *V*_cmax_ (*V*_cmax,25_) and *R*_d_ (*R*_d,25_) standardized to 25 °C. *V*_cmax,25_ was calculated using equation (5), with the *b* parameter set to 65 330 J mol^−1^, from Bernacchi *et al*. (2001). *R*_d,25_ was calculated using equation (2).

Relative extractable water (*θ*_R_) from the beginning of May (DOY 121) to the end of the experiment was analyzed using a mixed model analysis of variance with precipitation treatment, warming treatment, and their interaction as fixed factors. The experimental block and day of measurement (continuous) were considered random variables in the model. As precipitation treatments were nested within blocks and warming treatments were nested within precipitation treatments, these relationships were also included as random effects in the models.

Response variables *A*_n_, *R*_d_, *R*_d,25_, *E*, *g*_s_, *R/A*, *A*_n_*/g*_s_, *V*_cmax_, *V*_cmax,25_ and *l* were analyzed using mixed model analyses of variance with precipitation treatments, warming treatments and species as fixed effects and included all possible interactions. The experimental block, the individual, and the measurement week were included as random effects. Again, the nested relationships of precipitation treatments within blocks and warming treatments within precipitation treatments were included as random effects in the models. All model fitting was done using the ‘lmer’ procedure in the ‘lme4’ package (Bates *et al*. 2015) in R (R-Development-Core-Team 2009). Following model fitting, we calculated Wald *χ*^2^ statistics and performed type-II Wald tests for each fixed effect using the ‘Anova’ function in the ‘car’ package (Fox and Sanford 2011) in R. Least squared means were calculated using the ‘lsmeans’ function in the ‘lsmeans’ package (Lenth 2016) in R. *Post-hoc* comparison of means were done using Tukey’s Least Squared Difference tests using the ‘lsmeans’ package (Lenth, 2016) in R.

We also used structural equation modelling to examine the primary components directly and indirectly influencing *A*_n_ and *R*_d_. The analysis determined the components influencing *T*_leaf_, *E*, *D*_leaf_, *θ*_R_, *A*_n_, *g*_s_, *R*_d_ and *V*_cmax_. In addition to the predicted variables, the treatment types were used as explanatory variables. The path was determined using hypothesized relationships with a goal of capturing the primary determinants of *A*_n_ and *R*_d_. The precipitation treatment was converted to a continuous variable using rainfall amount (i.e. 0.5, 1, 1.5). Warming treatment was converted to a continuous variable using the heater wattage (i.e. 0, 200, 600, 1000). All variables were scaled before fitting the model. All species were included in the model. The path analysis was conducted using the ‘sem’ function in the ‘lavaan’ package (Rosseel 2012) in R. All analyses were performed using R version 3.2.1 (R-Development-Core-Team 2009).

## Results

### Treatment effects on soil moisture

**Figure 1.**
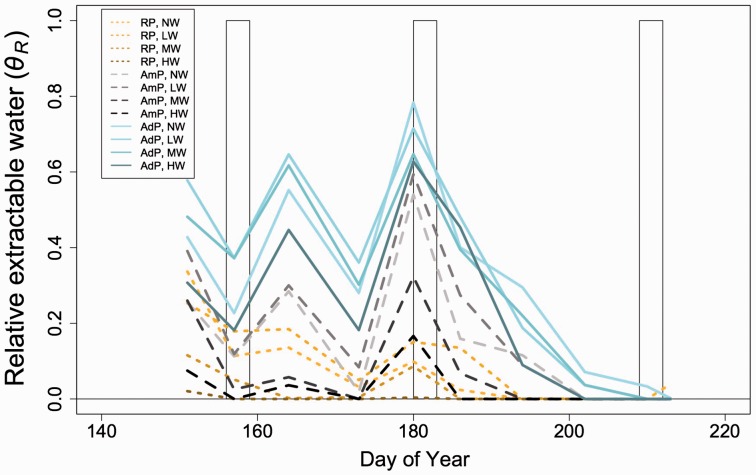
Mean relative extractable water (*θ*R; unitless) in the added precipitation (AdP; blue, solid lines), ambient precipitation (AmP; grey and black, dashed lines), and reduced precipitation (brown, dotted lines) over the course of the experiment. Darker colours within each precipitation treatment indicate higher levels of warming (NW, no warming; LW, low warming; MW,  medium warming; HW, high warming). Background bars indicate leaf gas exchange measurement periods. Means are for each plot type during each measurement date (*n = *3). Mixed model results related to this figure can be found in Table 1.

Both the warming (*P* < 0.01; Table 1) and precipitation (*P* < 0.01; Table 1) treatments had strong effects on *θ*_R_ in the plots. On average across the measurement dates, *θ*_R_ decreased by 27 % in the drought plots compared with ambient and increased by 61 % in the wet plots compared with the ambient plots. Warming increased *θ*_R_ by 9.5 % in the low warming plots, but decreased *θ*_R_ by 14 and 37 % in the medium and high warming plots, respectively. There was no interaction effect of warming and precipitation on *θ*_R_ (Fig. 1 and Table 1).

**Table 1.** Relative extractable water (*θ_R_*) mixed model results.

	Df	*χ^2^*	*P*-value
Precipitation (P)	2	17.01	**<0.001**
Warming (W)	3	13.03	**0.005**
P × W	6	0.99	0.986

*P*-values < 0.05 and 0.10 are bolded are bolded and italicized, respectively. Key: Df, degrees of freedom; *χ*^2^, Wald’s chi squared statistic.

### Leaf gas exchange responses to climate treatments

**Figure 2.**
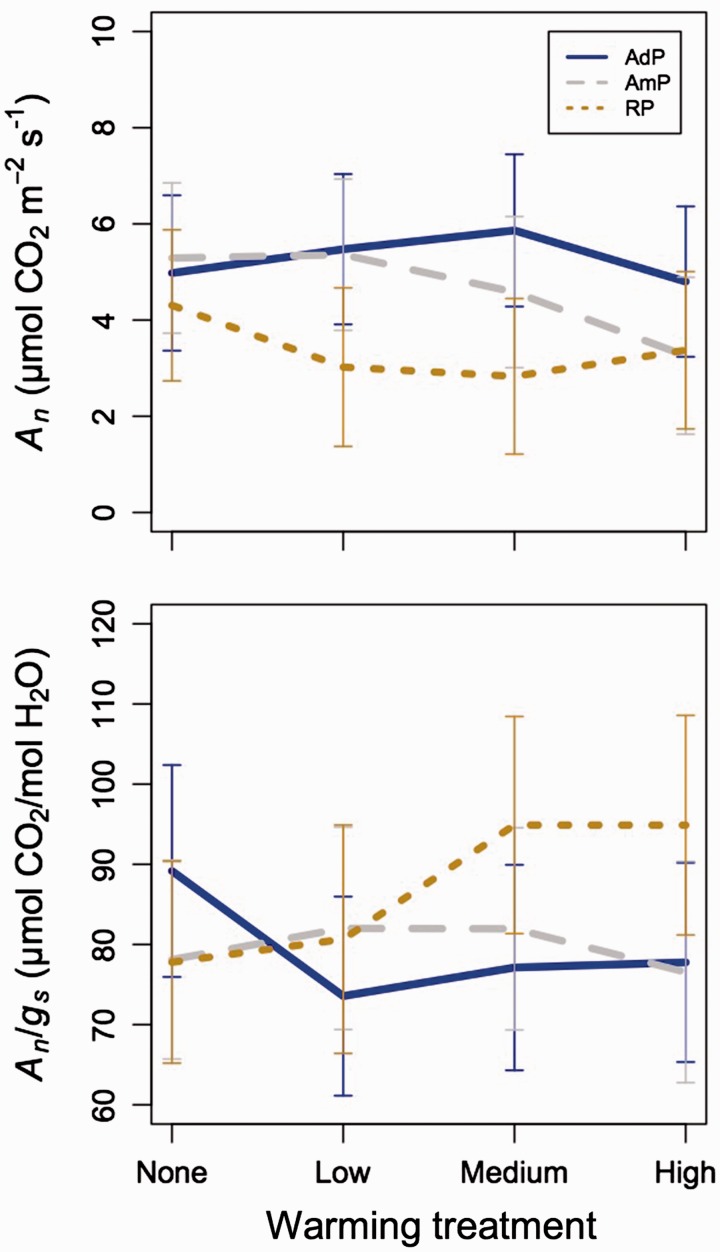
Least squared mean (±SE) net photosynthesis (*A*n; top) and intrinsic water use efficiency (*A*n/*g*s; bottom) in the added (AdP; blue, solid lines), ambient (AmP; grey, dashed lines), and reduced precipitation (brown, dotted lines) across each of the four warming treatments and all measurement dates and species. Mixed model results related to this figure can be found in Table 2.

Reduced precipitation significantly decreased net photosynthesis (*A*_n_) by 27 %, while added precipitation increased *A*_n_ by 14 % compared with ambient (*P* < 0.01; Fig. 2, Table 2, and **see** **Supporting Information**), effects consistent across all species (*P* > 0.05; Table 2). Warming had a marginally significant effect on *A*_n_ (*P* = * *0.062; Table 2), decreasing rates by 4.9, 8.9 and 22 % in the low, medium and high warming plots, respectively, compared with ambient (Fig. 2; Table 2). Gross photosynthesis (*A*_g_) responses to precipitation were similar to those of *A*_n_, decreasing by 21 % in response to reduced precipitation, and increasing by 11 % in response to added precipitation (*P* < 0.01; Table 2). Warming did not influence *A*_g_ (*P* > 0.05; Table 2). The maximum rate of Rubisco carboxylation, at ambient (*V*_cmax_) or standardized temperature (*V*_cmax,25_), did not respond significantly to the treatments or their interactions in any species (*P* > 0.05 in all cases; Table 2). The responses of *A*_n_, *A*_g_, *V*_cmax_ and *V*_cmax,25_ did not differ among species (*P* > 0.05 in all cases; Table 2).

**Table 2.** Mixed model results for parameters related to leaf CO_2_ uptake.

		*A*_n_		*A*_g_		*A*_n_*/g*_s_		*V*_cmax_		*V*_cmax,25_	
	Df	*χ^2^*	*P*-value	*χ^2^*	*P*-value	*χ^2^*	*P*-value	*χ^2^*	*P*-value	*χ^2^*	*P*-value
Precipitation (P)	2	9.62	**0.008**	17.48	**<0.001**	1.20	0.545	0.80	0.670	0.81	0.667
Warming (W)	3	7.33	*0.062*	3.80	0.284	4.08	0.253	3.35	0.341	3.67	0.299
Species (S)	4	59.07	**<0.001**	66.67	**<0.001**	150.12	**<0.001**	73.53	**<0.001**	57.10	**<0.001**
P × W	6	5.06	0.536	7.34	0.290	14.02	**0.029**	9.84	0.131	8.99	0.174
P × S	8	8.68	0.370	13.89	*0.085*	15.51	**0.0499**	9.08	0.336	6.48	0.594
W × S	12	13.52	0.332	14.66	0.260	9.14	0.691	9.43	0.666	6.59	0.883
P × W × S	24	22.19	0.568	15.83	0.894	14.77	0.927	21.79	0.592	18.55	0.775

*P*-values < 0.05 and 0.10 are bolded are bolded and italicized, respectively. Key: Df, degrees of freedom; *χ*^2^, Wald’s chi squared statistic; *A*_n_, net photosynthesis; *A*_g_, gross photosynthesis; *g*_s_, stomatal conductance; *V*_cmax_, maximum rate of Rubisco carboxylation at the ambient (i.e. measurement) leaf temperature, *V*_cmax,25_, maximum rate of Rubisco carboxylation standardized to a leaf temperature of 25 °C.

**Figure 3.**
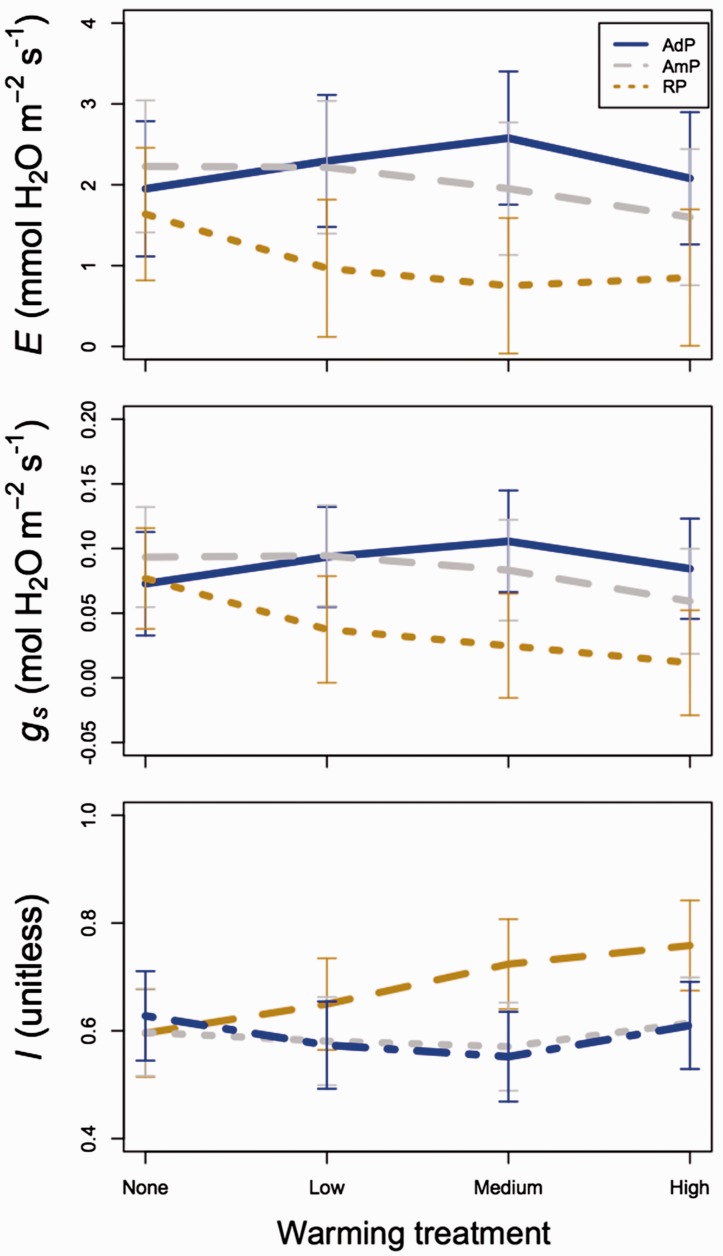
Least squared mean (±SE) transpiration (*E*; top), stomatal conductance (*g*s; middle) and stomatal limitation to photosynthesis (*l*; bottom) in the added (AdP; blue, solid lines), ambient (AmP; grey, dashed lines) and reduced precipitation (brown, dotted lines) across each of the four warming treatments and all measurement dates and species. Mixed model results related to this figure can be found in Table 3.

Transpiration (*E*) increased with increasing precipitation (*P* < 0.01) and decreased marginally with warming (*P* < 0.10; Table 3). There was no interaction between precipitation and warming (*P* > 0.05; Fig. 3 and Table 3) and treatment effects did not vary by species (*P* > 0.05 in all cases; Table 3).

**Table 3.** Mixed model results for parameters related to leaf transpiration and stomatal conductance

		*E*		*g*_s_		*l*	
	Df	*χ*^2^	*P*-value	*χ*^2^	*P*-value	*χ*^2^	*P*-value
Precipitation (P)	2	18.84	**<0.001**	23.03	**<0.001**	4.30	0.116
Warming (W)	3	6.38	*0.095*	11.91	**0.008**	24.21	**<0.001**
Species (S)	4	155.83	**<0.001**	109.30	**<0.001**	281.94	**<0.001**
P × W	6	7.41	0.284	16.21	**0.013**	36.71	**<0.001**
P × S	8	3.99	0.858	4.25	0.834	2.64	0.955
W × S	12	15.26	0.223	12.10	0.438	19.31	*0.081*
P × W × S	24	18.14	0.796	20.28	0.681	21.35	0.618

*P*-values < 0.05 and 0.10 are bolded are bolded and italicized, respectively. Key: Df, degrees of freedom, *χ*^2^, Wald’s chi squared statistic; *E*, transpiration; *g*_s_, stomatal conductance; *l*, stomatal limitation to photosynthesis.

Stomatal conductance (*g*_s_) increased with increasing precipitation (*P *<* *0.01; Table 3) and decreased with increasing warming (*P* < 0.01; Table 3), particularly in the reduced precipitation plots (precipitation x warming interaction: *P* < 0.05; Fig 3 and Table 3). Stomatal limitation of photosynthesis (*l*) increased with increased warming in the reduced precipitation plots, resulting in *l* values that were higher under reduced, compared with ambient and added precipitation in the medium and high warming plots (precipitation x warming interaction: *P* < 0.05; Figure 3 and Table 3). Treatment effects on *l* did not differ significantly by species (*P* > 0.05 in all cases; Table 3).

**Figure 4.**
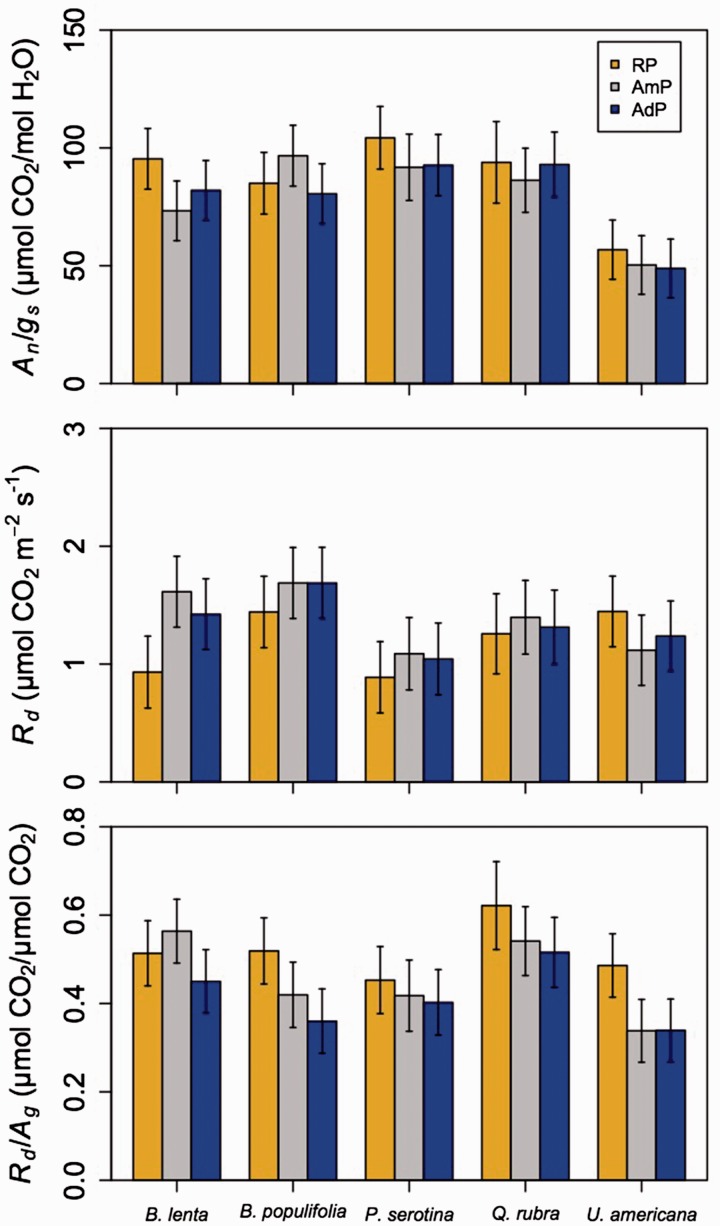
Least squared mean (±SE) ratio of net photosynthesis to stomatal conductance (*A*n/*g*s), leaf respiration in dark (*R*d), and the ratio of *R*d to gross photosynthesis (*R*d/*A*g) in each species in the reduced precipitation (brown), ambient (AmP; grey) and added (AdP; blue) precipitation plots. Mixed model results related to this figure can be found in Tables 2 (*A*n/*g*s) and 4 (*R*d and *R*d/*A*g). Results from related Tukey’s tests can be found in Table 5.

Intrinsic water use efficiency (*A*_n_/*g*_s_) increased with warming in the reduced precipitation plots, and decreased with warming in the added precipitation plots (precipitation × warming interaction: *P* < 0.05; Fig. 2 and Table 2). There was a weak precipitation by species effect (*P* *=* 0.0499; Fig. 4 and Table 2). *Post-hoc* analyses revealed that reduced precipitation alone marginally increased *A_n_/g_s_* in *B. lenta* compared with ambient precipitation (*P* *=* 0.058; Table 5). The within-species precipitation effect was not significant in any other case (*P* > 0.05 in all cases; Table 5).

Leaf dark respiration, at ambient (*R*_d_) and standardized (*R*_d,25_) temperatures, responded to altered precipitation in some, but not all, species (precipitation × species interaction: *P* < 0.01; Fig. 4, Table 4, and **see** **Supporting Information**). *Post-hoc* analyses revealed that *R*_d_ of *B. lenta* was lower under reduced (*P* < 0.05) and ambient (*P* < 0.05) compared with added precipitation, but was unaffected by precipitation in the other species (*P* > 0.05; Table 5). *Post-hoc* analyses indicated that *R*_d,25_ showed a similar response for *B. lenta* (Table 5). Interestingly, *R*_d,25_ of *U. americana* was significantly and marginally higher in the reduced compared with ambient (*P* < 0.05) and added (*P* = * *0.093) precipitation plots, respectively (Table 5). *R*_d,25_ was unaffected by altered precipitation in the other species (*P* > 0.05 in all cases; Table 5).

**Table 4.** Mixed model results for parameters related to leaf respiration.

	Df	*R*_d_		*R*_d,25_		*R*_d_*/A*_g_	
		*χ*^2^	*P*-value	*χ^2^*	*P*-value	*χ^2^*	*P*-value
Precipitation (P)	2	6.10	**0.047**	4.77	*0.092*	8.33	**0.016**
Warming (W)	3	2.11	0.549	1.43	0.700	12.17	**0.007**
Species (S)	4	45.03	**<0.001**	45.60	**<0.001**	52.52	**<0.001**
P × W	6	9.15	0.165	8.92	0.178	6.04	0.419
P × S	8	25.06	**0.002**	31.03	**<0.001**	15.61	**0.048**
W × S	12	12.60	0.399	7.29	0.838	9.33	0.674
P × W × S	24	16.78	0.858	18.60	0.773	22.81	0.531

*P*-values < 0.05 and 0.10 are bolded are bolded and italicized, respectively. Key: Df, degrees of freedom; *χ^2^*, Wald’s chi squared statistic; *R*_d_, leaf dark respiration at the ambient (i.e. measurement) leaf temperature, *R*_d,25_, leaf dark respiration standardized to a leaf temperature of 25 °C, *A*_g_, gross photosynthesis.

**Table 5.** Results from precipitation by species interaction tukey’s tests.

Response	Species	Contrast	Estimate	SE	Df	*t*-value	*P*-value
*A*_n_*/g*_s_	*B. lenta*	RP-AmP	*22.07*	*8.93*	*18.4*	*2.47*	*0.058*
		RP-AdP	13.35	8.90	17.9	1.50	0.315
		AmP-AdP	−8.72	8.58	15.9	−1.02	0.577
	*B. populifolia*	RP-AmP	−11.70	9.57	24.0	−1.22	0.452
		RP-AdP	4.47	9.37	21.8	0.48	0.883
		AmP-AdP	16.17	9.10	19.6	1.78	0.203
	*P. serotina*	RP-AmP	12.52	11.31	45.6	1.11	0.515
		RP-AdP	11.58	9.96	27.7	1.16	0.485
		AmP-AdP	−0.94	11.01	40.7	−0.09	0.996
	*Q. rubra*	RP-AmP	7.60	15.45	126.5	0.49	0.875
		RP-AdP	0.92	15.57	128.7	0.06	0.998
		AmP-AdP	−6.68	11.30	45.0	−0.59	0.825
	*U. americana*	RP-AmP	6.50	8.15	13.1	0.80	0.711
		RP-AdP	7.94	8.14	13.0	0.98	0.605
		AmP-AdP	1.43	7.93	11.8	0.18	0.982
*R*_d_	*B. lenta*	RP-AmP	**−0.68**	**0.16**	**50.7**	**−4.19**	<**0.001**
		RP-AdP	**−0.49**	**0.16**	**49.0**	**−3.01**	**0.011**
		AmP-AdP	0.19	0.16	43.6	1.23	0.443
	*B. populifolia*	RP-AmP	**−**0.25	0.16	47.2	**−**1.54	0.282
		RP-AdP	**−**0.25	0.16	48.3	**−**1.52	0.292
		AmP-AdP	0.00	0.16	46.8	0.00	1.000
	*P. serotina*	RP-AmP	**−**0.20	0.17	57.8	**−**1.18	0.471
		RP-AdP	**−**0.16	0.17	50.6	**−**0.93	0.621
		AmP-AdP	0.04	0.17	61.0	0.26	0.965
	*Q. rubra*	RP-AmP	**−**0.14	0.24	160.0	**−**0.59	0.827
		RP-AdP	**−**0.06	0.24	160.4	**−**0.23	0.970
		AmP-AdP	0.08	0.20	104.9	0.42	0.907
	*U. americana*	RP-AmP	*0.33*	*0.15*	*36.4*	*2.23*	*0.079*
		RP-AdP	0.21	0.15	34.4	1.44	0.334
		AmP-AdP	**−**0.12	0.14	32.7	**−**0.84	0.682
*R_d,25_*	*B. lenta*	RP-AmP	**−0.90**	**0.21**	**75.8**	**−4.37**	**<0.001**
		RP-AdP	**−0.58**	**0.21**	**71.3**	**−2.79**	**0.018**
		AmP-AdP	0.32	0.20	65.3	1.66	0.229
	*B. populifolia*	RP-AmP	**−**0.13	0.20	69.9	**−**0.66	0.790
		RP-AdP	**−**0.20	0.21	70.2	**−**0.96	0.605
		AmP-AdP	**−**0.06	0.20	69.9	**−**0.32	0.945
	*P. serotina*	RP-AmP	**−**0.24	0.22	85.1	**−**1.11	0.513
		RP-AdP	**−**0.21	0.21	71.6	**−**1.02	0.569
		AmP-AdP	0.02	0.22	87.0	0.11	0.994
	*Q. rubra*	RP-AmP	**−**0.33	0.31	212.4	**−**1.07	0.536
		RP-AdP	**−**0.42	0.31	211.3	**−**1.34	0.374
		AmP-AdP	**−**0.09	0.26	156.3	**−**0.34	0.937
	*U. americana*	RP-AmP	**0.55**	**0.19**	**54.4**	**2.97**	**0.012**
		RP-AdP	*0.39*	*0.18*	*51.1*	*2.14*	*0.093*
		AmP-AdP	**−**0.16	0.18	48.6	**−**0.89	0.650
*R_d_/A_g_*	*B. lenta*	RP-AmP	**−**0.05	0.05	19.1	**−**0.97	0.602
		RP-AdP	0.06	0.05	18.6	1.24	0.444
		AmP-AdP	*0.11*	*0.05*	*16.3*	*2.31*	*0.083*
	*B. populifolia*	RP-AmP	0.10	0.06	25.6	1.79	0.194
		RP-AdP	**0.16**	**0.05**	**23.5**	**2.92**	**0.020**
		AmP-AdP	0.06	0.05	21.6	1.12	0.512
	*P. serotina*	RP-AmP	0.04	0.07	47.2	0.54	0.852
		RP-AdP	0.05	0.06	28.6	0.88	0.657
		AmP-AdP	0.02	0.06	42.1	0.24	0.969
	*Q. rubra*	RP-AmP	0.08	0.09	131.5	0.90	0.642
		RP-AdP	0.11	0.09	133.2	1.17	0.471
		AmP-AdP	0.03	0.07	48.0	0.39	0.921
	*U. americana*	RP-AmP	**0.15**	**0.05**	**14.0**	**3.13**	**0.019**
		RP-AdP	**0.15**	**0.05**	**13.5**	**3.13**	**0.020**
		AmP-AdP	<0.01	0.05	12.4	**−**0.02	1.000

Contrast indicates the two treatments being compared within each species. Estimates are least squared mean differences in the response variable between the two treatments. SE are standard errors of the least squared mean. *P*-values < 0.05 and 0.10 are bolded are bolded and italicized, respectively. Key: Df, degrees of freedom; RP, RP; AmP, ambient precipitation; AdP, added precipitation; *A*_n_, net photosynthesis; *g*_s_, stomatal conductance; *R*_d_, leaf dark respiration at the ambient (i.e. measurement) leaf temperature; *R*_d,25_, leaf dark respiration standardized to a leaf temperature of 25 °C; *A*_g_, gross photosynthesis.

**Figure 5.**
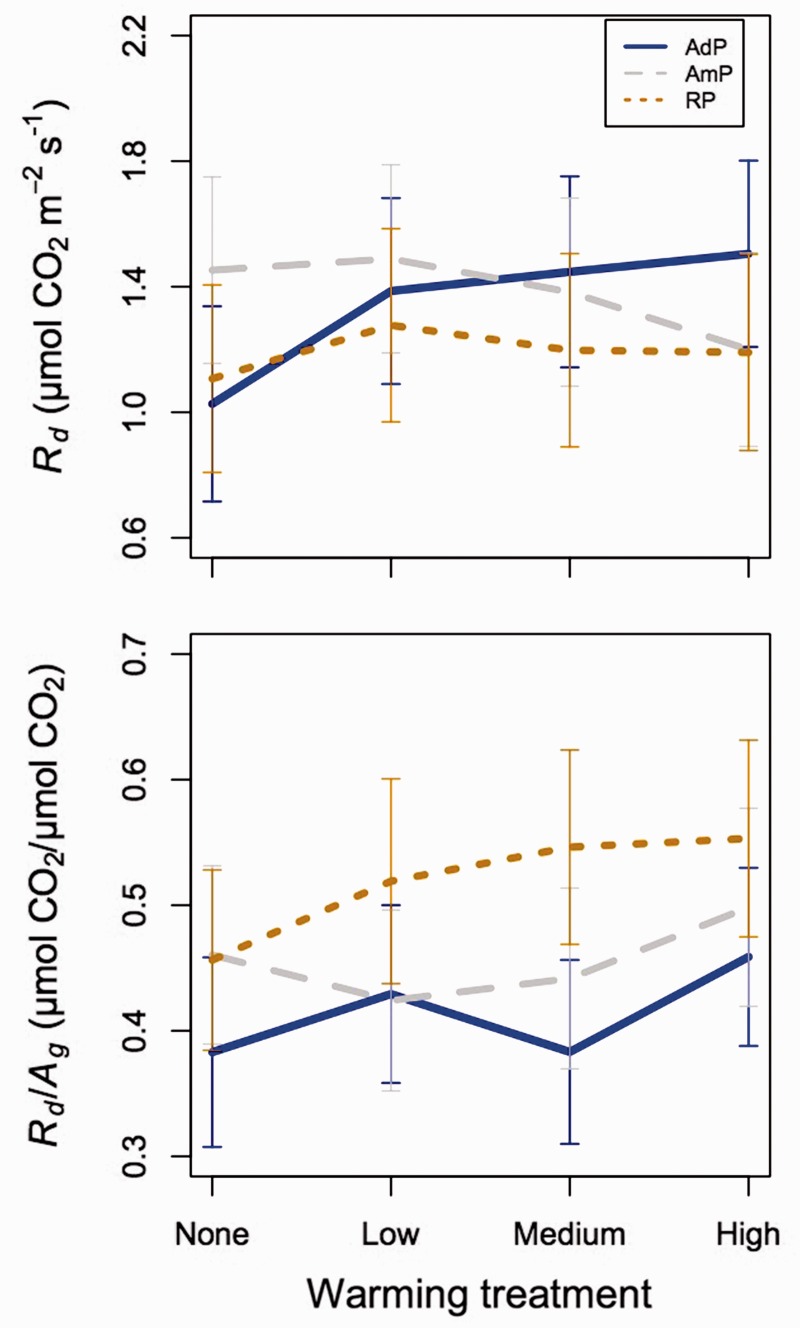
Least squared mean (±SE) dark leaf respiration (*R*d; top) and ratio of dark leaf respiration to gross photosynthesis (*R*d/*A*g; bottom) in the added (AdP; blue, solid lines), ambient (AmP; grey, dashed lines) and reduced precipitation (brown, dotted lines) across each of the four warming treatments and all measurement dates and species. Mixed model results related to this figure can be found in Table 4.

The ratio of *R*_d_ to *A*_g_ (*R*_d_/*A*_g_) increased by 5.6, 5.5 and 16 % in low, medium and high warming plots, respectively, compared with the ambient warming plots (*P* < 0.01; Fig. 5 and Table 4). Reduced and added precipitation increased *R*_d_/*A*_g_ by 14 % and decreased *R*_d_/*A*_g_ by 9.3 %, respectively, compared with the ambient precipitation plots (*P* < 0.05; Fig. 5 and Table 4). There was a weak precipitation by species interaction (*P* = * *0.048; Fig. 4 and Table 4). *Post-hoc* analyses revealed that the strongest increase in *R*_d_/*A*_g_ with decreased precipitation was observed in *B. populifolia* and *U. americana* (Fig. 4 and Table 5).

### Path analysis

**Figure 6.**
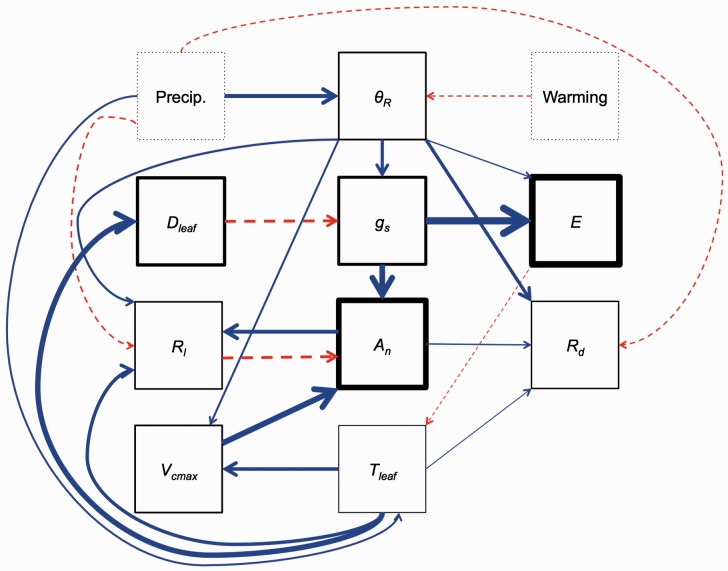
Path resulting from the structural equation modeling described in the text. Solid blue and dashed red arrows denote significant (*α* = 0.05) positive and negative relationships, respectively. The size of the arrow is indicative of the strength of the relationship. Non-significant relationships are not shown. The widths of the box outlines are related to the r2 value for each parameter assessed. Boxes outlined in dots indicate variables not predicted by the model. Key: Precip., precipitation treatment; Warming, warming treatment; *θ*R, relative extractable water; *E*, leaf transpiration, *D*leaf, leaf vapour pressure deficit; *g*s, stomatal conductance; *V*cmax, maximum rate of Rubisco carboxylation; *A*_n_, net photosynthesis; *R_l_*, leaf respiration in light; *R*_d_, leaf respiration in dark and *T*_leaf_, leaf temperature. Linear coefficient values, *Z* values, *P*-values, and *r*^2^ values for all parameters and relationships tested can be found in Table 6.

The structural equation modelling was designed to indicate the pathways, indirect and direct, by which the precipitation and warming treatments impacted leaf *A*_n_ and *R*_d_. *A*_n_ did not respond directly to the treatments (*P* > 0.05 in both cases; Table 6), but rather responded indirectly via responses to *g*_s_ (positive; *P* < 0.01; Table 6), *V*_cmax_ (positive; *P* < 0.01; Table 6), and *R*_l_ (negative; *P* < 0.01; Table 6). Interestingly, *T*_leaf_ (or *D*_leaf_ in the case of *g*_s_) and *θ*_R_ determined each of these three factors (*P* < 0.05 in all cases; Table 6). *θ*_R_ was directly related to the climate manipulations (*P* < 0.05 in both cases; Fig. 6 and Table 6). However, *T*_leaf_ was poorly predicted by the climatic treatments (*r*^2 ^ = ^ ^0.077), increasing with greater precipitation (*P* < 0.05) and decreasing with greater *E*, but showing no response to warming (*P* > 0.05; Fig. 6 and Table 6).

**Table 6.** Results from structural equation modelling

Dependent variable (*r*^2^)	Independent variable	Standardized coefficient	*Z*-value	*P*-value
*θ*_R_ (0.189)	**Precipitation**	**0.43**	**11.358**	**<0.001**
	**Warming**	**−0.137**	**−3.608**	**<0.001**
*T*_leaf_ (0.077)	**Precipitation**	**0.209**	**5.105**	**<0.001**
	***E***	**−0.103**	**−2.218**	**0.027**
	Warming	0.017	0.423	0.672
*D*_leaf_ (0.360)	***T*_leaf_**	**0.583**	**17.077**	**<0.001**
*g*_s_ (0.327)	***θ*_R_**	**0.336**	**8.328**	**<0.001**
	***D*_leaf_**	**−0.309**	**−8.001**	**<0.001**
	*A*_n_	0.091	1.67	0.095
	Warming	−0.018	−0.518	0.604
	Precipitation	−0.007	−0.184	0.854
*E* (0.728)	***g*_s_**	**0.789**	**33.728**	**<0.001**
	***θ*_R_**	**0.14**	**6.021**	**<0.001**
*V*_cmax_ (0.188)	***T*_leaf_**	**0.383**	**9.953**	**<0.001**
	***θ*_R_**	**0.216**	**5.173**	**<0.001**
	Precipitation	−0.036	−0.837	0.403
	Warming	−0.025	−0.658	0.511
*R*_l_ (0.175)	***A*_n_**	**0.414**	**7.982**	**<0.001**
	***T*_leaf_**	**0.369**	**9.523**	**<0.001**
	***θ*_R_**	**0.237**	**5.385**	**<0.001**
	**Precipitation**	**−0.176**	**−4.071**	**<0.001**
	Warming	0.046	1.189	0.235
*A*_n_ (0.611)	***V*_cmax_**	**0.623**	**20.787**	**<0.001**
	***g*_s_**	**0.62**	**18.211**	**<0.001**
	***R*_l_**	**−0.306**	**−8.242**	**<0.001**
	Precipitation	−0.006	−0.213	0.831
	Warming	−0.001	−0.026	0.979
*R*_d_ (0.158)	***θ*_R_**	**0.361**	**8.256**	**<0.001**
	***A*_n_**	**0.147**	**3.69**	**<0.001**
	**Precipitation**	**−0.152**	**−3.549**	**<0.001**
	***T*_leaf_**	**0.094**	**2.429**	**0.015**
	Warming	0.073	1.877	0.06

Variable key: *θ*_R_, relative extractable water; *g*_s_, stomatal conductance; *V*_cmax_, maximum rate of Rubisco carboxylation; *R*_l_, leaf respiration in light; *A*_n_, net photosynthesis; *R*_d_, leaf respiration in dark; *D*_leaf_, leaf vapour pressure deficit; *T*_leaf_, leaf temperature. Independent variables are ordered by the absolute value of the standardized coefficient for each dependent variable.

In contrast, *R*_d_ was influenced both directly and indirectly by the climatic changes. The strongest determinant of *R*_d_ was *θ*_R_ (positive; *P* < 0.01; Table 6), but *T*_leaf_ (*P* < 0.01; Table 6) and *A*_n_ (*P* < 0.01; Table 6) also increased *R*_d_. Interestingly, the precipitation treatment had a direct negative influence on *R*_d_ (*P* < 0.01; Table 6), indicating that precipitation has differing influences on *R*_d_ depending on time scale (i.e. positive in short term, but negative in long term; Fig. 6 and Table 6). The warming treatment had a marginally significant positive influence on *R*_d_ (*P * = 0.060; Table 6), indicating that warming can have both short- and, to a lesser degree, long-term effects on *R*_d_.

## Discussion

We used a climate manipulation experiment in an old-field ecosystem to examine the responses of net photosynthesis (*A*_n_) and dark respiration (*R*_d_) to warming and altered precipitation. Our goal was to not only to examine the responses, but to also to probe the mechanisms underlying them. Confirming theoretical understanding (Lin *et al*. 2012), *A*_n_ was controlled by a combination of stomatal conductance (*g*_s_), leaf biochemistry (i.e. *V*_cmax_), and leaf respiration (i.e. *R*_l_). Leaf dark respiration was less sensitive to climate than *A*_n_, due to offsetting long- and short-term effects of soil moisture.

In accordance with our original hypothesis, the three primary determinants of *A_n_* were heavily influenced by direct (via altered precipitation) and indirect (via warming) soil moisture effects. Soil moisture significantly increased under increasing precipitation and decreased under warming over the course of our experiment. The precipitation response is not surprising. However, the warming response, while not as strong, indicates that warming-induced reductions in soil moisture may exacerbate the plant gas exchange responses to drought observed in precipitation manipulation-only studies (e.g. Wu *et al*. 2011; Yan *et al*. 2016). Reductions in soil moisture reduced *g*_s_ and *V*_cmax_, the limiting enzymatic process at the light levels assessed (Long and Bernacchi 2003), which would contribute to the reduced *A*_n_ seen under warming and lower precipitation. However, soil moisture also reduced *R*_l_, which would should have increased *A*_n_, but was a weaker driver than *g*_s_ and *V*_cmax_.

The leaf temperature at the time of measurement also influenced each of the three drivers of *A*_n_. Stomatal conductance decreased with increased vapour pressure deficit (*D*_leaf_), which is positively correlated with leaf temperature. The enzymatic processes *V*_cmax_ and *R*_l_ accelerated with increasing leaf temperature, as has been seen in many studies (e.g. Ryan 1991; Bernacchi *et al*. 2001; Medlyn *et al*. 2002a,b; Atkin *et al*. 2005). These results confirm theoretical understanding (Lin *et al*. 2012). Nonetheless, in contrast with our expectations, warming did not directly influence leaf temperatures at the time of measurement due to the measurement cuvette blocking incoming thermal radiation from the heaters. Instead, warming-induced reductions in soil moisture reduced transpiration, which led to higher leaf temperatures.

Our results provide insight for model development of moisture-stomata-photosynthesis relationships. Currently, many models simulate photosynthesis and stomatal conductance as interdependent, instantaneous responses (e.g. through coupled schemes; Ball *et al*. 1987; Collatz *et al*. 1991, 1992; Leuning 1995; Medlyn *et al*. 2011) in which *g*_s_ is a function of *A*_n_ and *D*_leaf_ (or relative humidity), while *A*_n_ is typically simulated as function of light, *T*_leaf_, soil moisture, and other leaf traits such as leaf N and leaf age. Our path analysis of field data (Fig. 5) found that moisture and *D*_leaf_ were the primary drivers of *g*_s_, which, in turn, drove *A*_n_ (along with leaf temperature). *A*_n_ was not found to be a significant driver of *g*_s_. This result indicates that *A*_n_ might be best simulated as a function of *g*_s_, rather than separately as a function of a coupled scheme.

In addition, we found significant changes in the *A*_n_/*g*_s_ ratio with climate. This result implies that models should consider including climatic responses as part of the coupled *A*_n_–*g*_s_ scheme. However, more model-data comparisons at the leaf (e.g. Egea *et al*. 2011) and larger scales (e.g. Keenan *et al*. 2010; De Kauwe *et al*. 2013) are necessary to fully evaluate these responses.

Our climate treatments had a less pronounced effect on *R*_d_ than *A*_n_. Precipitation increased *R*_d_ and *R*_d,25_ in the *B. lenta* and decreased *R*_d,25_ in *U. americana*, but had little effect on *R*_d_ of other species. Warming had little effect in general, which contrasts with results of a recent warming-only study examining leaf respiration in similar species (Reich *et al*. 2016). Our path analysis allowed us to gain some insight into the drivers of *R*_d_ responses to climate over varying time scales. In the short term, increased photosynthesis, temperature, and soil moisture acted to increase *R*_d_. This temperature response is widely observed (Atkin *et al*. 2005) and, given that photosynthesis provides the substrate for respiration (Gifford, 2003), it is not surprising that higher *A*_n_ would lead to higher *R*_d_. The positive response to soil moisture may indicate an increase in growth demand for respiratory products under more favourable (i.e. wetter) conditions. Of interest, and counter to the soil moisture response, was the observed increase in *R*_d_ with decreasing amounts of precipitation. This result may reflect a longer-term response to prolonged drought stress that increased the demand for maintenance respiration, as has been seen in other studies (Gratani *et al*. 2007; Slot *et al*. 2008; Atkin and Macherel 2009). A combination of offsetting long- and short-term responses likely contributed to the weak response observed when considering the climate treatments alone. These results suggest that the effect of soil moisture on *R*_d_ varies with the duration of exposure to a given soil moisture level. This time dependency of the soil moisture response may complicate the interpretation of the effects of other treatments such as warming (e.g. Reich *et al*. 2016), particularly when those other treatments have consequences for soil moisture. Targeted measurements within multi-factor experiments could help to pinpoint the mechanisms underlying respiration responses to climate.

Combined with the photosynthesis results, the weak response of respiration resulted in a decrease in the ratio of dark respiration to gross photosynthesis (*R*_d_/*A*_g_) under cooler and wetter conditions. Similar precipitation results have been seen in response to seasonal change in soil moisture in Mediterranean species (Gratani *et al*. 2008); however, the response of the respiration-photosynthesis relationship to water availability is understudied in the field outside of the Mediterranean region (Chaves *et al*. 2002, 2009; Pinheiro and Chaves 2011). Our results suggest that, for seedlings in the northeastern USA, future net leaf carbon uptake will likely decrease unless precipitation can raise soil moisture levels enough to counteract the negative effects of warming on soil moisture. However, as we were not able to measure the respiration of other tissues, we cannot address the whole-plant biosphere-atmosphere interaction.

We expected that the *Betula* species would be most sensitive to our climate manipulations due to their relatively restricted current and projected ranges (Prasad *et al*. 2007-ongoing; Iverson *et al*. 2008). Although the magnitude of gas exchange rates did differ by species, there were very few species-specific treatment responses. Nonetheless, *Betula* was generally more sensitive to soil moisture than other species. For instance, while all species tended to increase *A*_n_/*g*_s_ in response to a combination of hotter and drier conditions, *B. lenta* increased this ratio in response to reduced precipitation alone, an effect seen in previous studies on other *Betula* species (Wang *et al*. 1998). This indicates that *B. lenta* may have a more risk-averse drought strategy than the other species examined here. Similarly, *B. populifolia*, *U. americana*, and, to a lesser degree, *B. lenta* showed the greatest sensitivity of *R*_d_/*A*_g_ to precipitation. In these species, *R*_d_/*A*_g_ rates increased with drought, indicating a risk-averse switch in resources from carbon acquisition to maintenance and growth of tissues. Although there were notable similarities among species examined, the few species-specific differences that did exist suggest that further examination of a more diverse range of plant species, particularly those that exist in narrow climatic ranges or at range edges (Reich *et al*. 2015), may reveal more differential responses.

## Conclusions

Our findings indicate that warming and altered precipitation influence leaf carbon exchange primarily through indirect effects on environmental conditions and underlying processes. Leaf respiration was less sensitive than photosynthesis to our treatments, indicating that net leaf carbon uptake was increasing in wetter and, to a lesser degree, cooler conditions. As such, the net effect of climate change on carbon uptake in these species is likely to depend on whether the benefits associated with any precipitation increases can counteract the negative effects of temperature increases. More analyses, particularly of respiration responses in stems, roots and soil, are needed to explore the influence of these findings on ecosystem-level carbon uptake. These data could prove useful for evaluating the larger-scale models used to make future projections of climate-carbon feedbacks and, to facilitate such use, are included in the TRY (www.try-db.org) and open-access Purdue University Research Repository (https://purr.purdue.edu/publications/2213/2). 

## Sources of Funding

Our work was funded by the National Science Foundation (DEB-0546670 and DEB-1146279), US Department of Energy’s Office of Science (BER), through the Northeastern Regional Center of the National Institute for Climatic Change Research, National Aeronautics and Space Administration (NNX13AN35H), US Department of Agriculture (2015-67003-23485), and the Purdue Climate Change Research Center. 

## Contributions by the Authors

N.G.S. and J.S.D. conceived of the project. N.G.S., G.P. and C.E.G. took the measurements. N.G.S. analysed the data. All authors contributed to the writing of the article.

## Conflicts of Interest Statement

None declared.

## Supplementary Material

Supplementary Data

## Supporting Information

The following additional information is available in the online version of this article — **Figure S1.** Raw net photosynthesis (An) and leaf dark respiration (Rd) data for all species, treatments, and days of year.

## References

[plw066-B1] Arneth A Harrison SP Zaehle S Tsigaridis K Menon S Bartlein PJ Feichter J Korhola A Kulmala M O'Donnell D Schurgers G Sorvari S Vesala T. 2010 Terrestrial biogeochemical feedbacks in the climate system. Nature Geoscience 3:525–532.

[plw066-B2] Atkin OK Bruhn D Hurry VM Tjoelker MG. 2005 The hot and the cold: unravelling the variable response of plant respiration to temperature. Functional Plant Biology 32:87–105. 10.1071/FP03176 32689114

[plw066-B3] Atkin OK Macherel D. 2009 The crucial role of plant mitochondria in orchestrating drought tolerance. Annals of Botany 103:581–597. 18552366 10.1093/aob/mcn094 PMC2707344

[plw066-B4] Ball JT Woodrow I Berry J. 1987 A model predicting stomatal conductance and its contribution to the control of photosynthesis under different environmental conditions In: Biggins J, ed. Progress in Photosynthesis Research. Netherlands: Springer.

[plw066-B5] Bates D Mächler M Bolker B Walker S. 2015 Fitting linear mixed-effects models using lme4. Journal of Statistical Software 67: 1-48.

[plw066-B6] Bernacchi CJ Singsaas EL Pimentel C Portis AR Long SP. 2001 Improved temperature response functions for models of Rubisco-limited photosynthesis. Plant Cell and Environment 24:253–259.

[plw066-B7] Camberlin P Martiny N Philippon N Richard Y. 2007 Determinants of the interannual relationships between remote sensed photosynthetic activity and rainfall in tropical Africa. Remote Sensing of Environment 106:199–216.

[plw066-B8] Chaves M Flexas J Pinheiro C. 2009 Photosynthesis under drought and salt stress: regulation mechanisms from whole plant to cell. Annals of Botany 103:551–560. 18662937 10.1093/aob/mcn125 PMC2707345

[plw066-B9] Chaves MM Pereira JS Maroco J Rodrigues ML Ricardo CPP Osorio ML Carvalho I Faria T Pinheiro C. 2002 How plants cope with water stress in the field? Photosynthesis and growth. Annals of Botany 89:907–916. 12102516 10.1093/aob/mcf105 PMC4233809

[plw066-B10] Collatz GJ Ball JT Grivet C Berry JA. 1991 Physiological and environmental regulation of stomatal conductance, photosynthesis, and transpiration - a model that includes a laminar boundary layer. Agricultural and Forest Meteorology 54:107–136.

[plw066-B11] Collatz GJ Ribas-Carbo M Berry JA. 1992 Coupled photosynthesis-stomatal conductance model for leaves of C4 plants. Australian Journal of Plant Physiology 19:519–538.

[plw066-B12] De Kauwe MG Lin Y-S Wright IJ Medlyn BE Crous KY Ellsworth DS Maire V Prentice IC Atkin OK Rogers A Niinemets Ü Serbin SP Meir P Uddling J Togashi HF Tarvainen L Weerasinghe LK Evans BJ Ishida FY Domingues TF. 2015 A test of the ‘one-point method’ for estimating maximum carboxylation capacity from field-measured, light-saturated photosynthesis. New Phytologist 120:1130–1144. 10.1111/nph.13815 26719951

[plw066-B13] De Kauwe MG Medlyn BE Zaehle S Walker AP Dietze MC Hickler T Jain AK Luo Y Parton WJ Prentice C Smith B Thornton PE Wang S Wang Y-P Wårlind D Weng ES Crous KY Ellsworth DS Hanson PJ Seok-Kim H Warren JM Oren R Norby RJ. 2013 Forest water use and water use efficiency at elevated CO_2_: a model-data intercomparison at two contrasting temperate forest FACE sites. Global Change Biology 19:1759–1779. 23504858 10.1111/gcb.12164

[plw066-B14] Egea G Verhoef A Vidale PL. 2011 Towards an improved and more flexible representation of water stress in coupled photosynthesis–stomatal conductance models. Agricultural and Forest Meteorology 151:1370–1384.

[plw066-B15] Farquhar G von Caemmerer S Berry J. 1980 A biochemical model of photosynthetic CO_2_ assimilation in leaves of C_3_ species. Planta 149:78–90. 24306196 10.1007/BF00386231

[plw066-B16] Farquhar GD Sharkey TD. 1982 Stomatal Conductance and Photosynthesis. Annual Review of Plant Physiology 33:317–345.

[plw066-B17] Flexas J Bota J Galmes J Medrano H Ribas-Carbo M. 2006 Keeping a positive carbon balance under adverse conditions: responses of photosynthesis and respiration to water stress. Physiologia Plantarum 127:343–352.

[plw066-B18] Fox J Sanford W. 2011 An {R} Companion to Applied Regression. Thousand Oaks, CA: Sage.

[plw066-B19] Friedlingstein P Meinshausen M Arora VK Jones CD Anav A Liddicoat SK Knutti R. 2013 Uncertainties in CMIP5 Climate Projections due to Carbon Cycle Feedbacks. Journal of Climate 27:511–526.

[plw066-B20] Galmes J Ribas-Carbo M Medrano H Flexas J. 2007 Response of leaf respiration to water stress in Mediterranean species with different growth forms. Journal of Arid Environments 68:206–222.

[plw066-B21] Gifford RM. 2003 Plant respiration in productivity models: conceptualisation, representation and issues for global terrestrial carbon-cycle research. Functional Plant Biology 30:171–186. 10.1071/FP02083 32689003

[plw066-B22] Gimeno TE Sommerville KE Valladares F Atkin OK. 2010 Homeostasis of respiration under drought and its important consequences for foliar carbon balance in a drier climate: insights from two contrasting *Acacia* species. Functional Plant Biology 37:323–333.

[plw066-B23] Gratani L Varone L Bonito A. 2007 Environmental induced variations in leaf dark respiration and net photosynthesis of Quercus ilex L. Photosynthetica 45:633–636.

[plw066-B24] Gratani L Varone L Catoni R. 2008 Relationship between net photosynthesis and leaf respiration in Mediterranean evergreen species. Photosynthetica 46:567–573.

[plw066-B25] Harte J Torn MS Chang F-R Feifarek B Kinzig AP Shaw R Shen K. 1995 Global warming and soil microclimate: results from a meadow-warming experiment. Ecological Applications 5:132–150.

[plw066-B26] Hayhoe K Wake C Huntington T Luo L Schwartz M Sheffield J Wood E Anderson B Bradbury J DeGaetano A Troy T Wolfe D. 2007 Past and future changes in climate and hydrological indicators in the US Northeast. Climate Dynamics 28:381–407.

[plw066-B27] Hoeppner SS Dukes JS. 2012 Interactive responses of old-field plant growth and composition to warming and precipitation. Global Change Biology 18:1754–1768.

[plw066-B28] Hsiao TC. 1973 Plant Responses to Water Stress. Annual Review of Plant Physiology 24:519–570.

[plw066-B29] Ignace DD Huxman TE Weltzin JF Williams DG. 2007 Leaf gas exchange and water status responses of a native and non-native grass to precipitation across contrasting soil surfaces in the Sonoran Desert. Oecologia 152:401–413. 17333286 10.1007/s00442-007-0670-x

[plw066-B30] IPCC. 2013 Climate Change 2013: The Physical Science Basis. Contribution of Working Group I to the Fifth Assessment Resport of the Intergovernmental Panel on Climate Change. New York, NY: Cambridge University Press.

[plw066-B31] Iverson LR Prasad AM Matthews SN Peters M. 2008 Estimating potential habitat for 134 eastern US tree species under six climate scenarios. Forest Ecology and Management 254:390–406.

[plw066-B32] Keenan T Sabate S Gracia C. 2010 Soil water stress and coupled photosynthesis–conductance models: bridging the gap between conflicting reports on the relative roles of stomatal, mesophyll conductance and biochemical limitations to photosynthesis. Agricultural and Forest Meteorology 150:443–453.

[plw066-B33] Kroner Y Way DA. 2016 Carbon fluxes acclimate more strongly to elevated growth temperatures than to elevated CO_2_ concentrations in a northern conifer. Global Change Biology: N/a-N/a10.1111/gcb.13215 26728638

[plw066-B34] Le Quéré C Andres RJ Boden T Conway T Houghton RA House JI Marland G Peters GP van der Werf G Ahlström A Andrew RM Bopp L Canadell JG Ciais P Doney SC Enright C Friedlingstein P Huntingford C Jain AK Jourdain C Kato E Keeling RF Klein Goldewijk K Levis S Levy P Lomas M Poulter B Raupach MR Schwinger J Sitch S Stocker BD Viovy N Zaehle S Zeng N. 2012 The global carbon budget 1959–2011. Earth System Science Data Discussions 5:1107–1157.

[plw066-B35] Lenth R. 2016 Least-squares Means: the R Package lsmeans. Journal of Statitstical Software 69:1–33.

[plw066-B36] Leuning R. 1995 A critical appraisal of a combined stomatal-photosynthesis model for C_3_ plants. Plant, Cell and Environment 18:339–355.

[plw066-B37] Li YG Jiang GM Liu MZ Niu SL Gao LM Cao XC. 2007 Photosynthetic response to precipitation/rainfall in predominant tree (*Ulmus pumila*) seedlings in Hunshandak Sandland, China. Photosynthetica 45:133–138.

[plw066-B38] Lin Y-S Medlyn BE Ellsworth DS. 2012 Temperature responses of leaf net photosynthesis: the role of component processes. Tree Physiology 32:219–231. 22278379 10.1093/treephys/tpr141

[plw066-B39] Llorens L Penuelas J Beier C Emmett B Estiarte M Tietema A. 2004 Effects of an experimental increase of temperature and drought on the photosynthetic performance of two ericaceous shrub species along a north-south European gradient. Ecosystems 7:613–624.

[plw066-B40] Long SP Bernacchi CJ. 2003 Gas exchange measurements, what can they tell us about the underlying limitations to photosynthesis? Procedures and sources of error. Journal of Experimental Botany 54:2393–2401. 14512377 10.1093/jxb/erg262

[plw066-B41] Lu M Zhou X Yang Q Li H Luo Y Fang C Chen J Yang X Li B. 2012 Responses of ecosystem carbon cycle to experimental warming: a meta-analysis. Ecology 94:726–738. 10.1890/12-0279.1 23687898

[plw066-B42] Medlyn BE Dreyer E Ellsworth D Forstreuter M Harley PC Kirschbaum MUF Le Roux X Montpied P Strassemeyer J Walcroft A Wang K Loustau D. 2002a Temperature response of parameters of a biochemically based model of photosynthesis. II. A review of experimental data. Plant Cell and Environment 25:1167–1179.

[plw066-B43] Medlyn BE Duursma RA Eamus D Ellsworth DS Prentice IC Barton CVM Crous KY De Angelis P Freeman M Wingate L. 2011 Reconciling the optimal and empirical approaches to modelling stomatal conductance. Global Change Biology 17:2134–2144.

[plw066-B44] Medlyn BE Loustau D Delzon S. 2002b Temperature response of parameters of a biochemically based model of photosynthesis. I. Seasonal changes in mature maritime pine (Pinus pinaster Ait.). Plant Cell and Environment 25:1155–1165.

[plw066-B46] Ocheltree TW Nippert JB Prasad PVV. 2014 Stomatal responses to changes in vapor pressure deficit reflect tissue-specific differences in hydraulic conductance. Plant, Cell and Environment 37:132–139. 10.1111/pce.12137 23701708

[plw066-B47] Oleson KW Lawrence DM Bonan GB Flanner MG Kluzek E Lawrence PJ Levis S Swenson SC Thornton PE Dai A Decker M Dickinson RE Feddema J Heald CL Hoffman FM Lamarque JF Mahowald N Niu G-Y Qian T Randerson J Running S Sakaguchi K Slater A Stockli R Wang A Yang Z-L Zeng X Zeng X. 2010 Technical description of version 4.0 of the Community Land Model (CLM). Boulder, CO: National Center for Atmospheric Research.

[plw066-B48] Pinheiro C Chaves MM. 2011 Photosynthesis and drought: can we make metabolic connections from available data? Journal of Experimental Botany 62:869–882. 21172816 10.1093/jxb/erq340

[plw066-B49] Potter CS Randerson JT Field CB Matson PA Vitousek PM Mooney HA Klooster SA. 1993 Terrestrial ecosystem production - a process model-based on global satellite and surface data. Global Biogeochemical Cycles 7:811–841.

[plw066-B50] Prasad AM Iverson LR Matthews SN Peters M. 2007-ongoing. A Climate Change Atlas for 134 Forest Tree Species of the Eastern United States [database]. Delaware, Ohio: Northern Research Station, USDA Forest Service.

[plw066-B51] R Core Team. 2015 R: A language and environment for statistical computing. R Foundation for Statistical Computing, Vienna, Austria. URL http://www.R-project.org/.

[plw066-B52] Reich PB Sendall KM Rice K Rich RL Stefanski A Hobbie SE Montgomery RA. 2015 Geographic range predicts photosynthetic and growth response to warming in co-occurring tree species. Nature Clim. Change 5:148–152.

[plw066-B53] Reich PB Sendall KM Stefanski A Wei X Rich RL Montgomery RA. 2016 Boreal and temperate trees show strong acclimation of respiration to warming. Nature 531:633–636. 26982730 10.1038/nature17142

[plw066-B54] Ribas-Carbo M Taylor NL Giles L Busquets S Finnegan PM Day DA Lambers H Medrano H Berry JA Flexas J. 2005 Effects of water stress on respiration in soybean leaves. Plant Physiology 139:466–473. 16126857 10.1104/pp.105.065565 PMC1203395

[plw066-B55] Rodgers VL Hoeppner SS Daley MJ Dukes JS. 2012 Leaf-level gas exchange and foliar chemistry of common old-field species responding to warming and precipitation treatments. International Journal of Plant Sciences 173:957–970.

[plw066-B56] Rosseel Y. 2012 lavaan: An R Package for Structural Equation Modeling. Journal of Statistical Software 48:1–36.

[plw066-B57] Ryan MG. 1991 A simple method for estimating gross carbon budgets for vegetation in forest ecosystems. Tree Physiology 9:255–266. 14972868 10.1093/treephys/9.1-2.255

[plw066-B58] Saxton KE Rawls WJ. 2006 Soil Water Characteristic Estimates by Texture and Organic Matter for Hydrologic Solutions. Soil Science Society of America Journal 70:1569–1578.

[plw066-B59] Seneviratne SI Corti T Davin EL Hirschi M Jaeger EB Lehner I Orlowsky B Teuling AJ. 2010 Investigating soil moisture–climate interactions in a changing climate: A review. Earth-Science Reviews 99:125–161.

[plw066-B60] Shaw B Thomas TH Cooke DT. 2002 Responses of sugar beet (Beta vulgaris L.) to drought and nutrient deficiency stress. Plant Growth Regulation 37:77–83.

[plw066-B61] Slot M Kitajima K. 2014 General patterns of acclimation of leaf respiration to elevated temperatures across biomes and plant types. Oecologia 177:885–900. 25481817 10.1007/s00442-014-3159-4

[plw066-B62] Slot M Zaragoza-Castells J Atkin OK. 2008 Transient shade and drought have divergent impacts on the temperature sensitivity of dark respiration in leaves of *Geum urbanum*. Functional Plant Biology 35:1135–1146. 10.1071/FP08113 32688861

[plw066-B63] Smith NG Rodgers VL Brzostek ER Kulmatiski A Avolio ML Hoover DL Koerner SE Grant K Jentsch A Fatichi S Niyogi D. 2014 Toward a better integration of biological data from precipitation manipulation experiments into Earth system models. Reviews of Geophysics 52:412–434.

[plw066-B64] Suseela V Conant RT Wallenstein MD Dukes JS. 2012 Effects of soil moisture on the temperature sensitivity of heterotrophic respiration vary seasonally in an old-field climate change experiment. Global Change Biology 18:336–348.

[plw066-B65] Tjoelker MG Oleksyn J Reich PB. 2001 Modelling respiration of vegetation: evidence for a general temperature-dependent Q(10). Global Change Biology 7:223–230.

[plw066-B66] Van Oijen M Schapendonk A Hoglind M. 2010 On the relative magnitudes of photosynthesis, respiration, growth and carbon storage in vegetation. Annals of Botany 105:793–797. 20237118 10.1093/aob/mcq039 PMC2859914

[plw066-B67] Vicca S Gilgen AK Camino Serrano M Dreesen FE Dukes JS Estiarte M Gray SB Guidolotti G Hoeppner SS Leakey ADB Ogaya R Ort DR Ostrogovic MZ Rambal S Sardans J Schmitt M Siebers M van der Linden L van Straaten O Granier A. 2012 Urgent need for a common metric to make precipitation manipulation experiments comparable. New Phytologist 195:518–522. 22734795 10.1111/j.1469-8137.2012.04224.x

[plw066-B68] Wang JR Hawkins CDB Letchford T. 1998 Photosynthesis, water and nitrogen use efficiencies of four paper birch (Betula papyrifera) populations grown under different soil moisture and nutrient regimes. Forest Ecology and Management 112:233–244.

[plw066-B69] Way DA Yamori W. 2014 Thermal acclimation of photosynthesis: on the importance of adjusting our definitions and accounting for thermal acclimation of respiration. Photosynthesis Research 119:89–100. 23812760 10.1007/s11120-013-9873-7

[plw066-B70] Wu Z Dijkstra P Koch GW Peñuelas J Hungate BA. 2011 Responses of terrestrial ecosystems to temperature and precipitation change: a meta-analysis of experimental manipulation. Global Change Biology 17:927–942.

[plw066-B71] Yamori W Hikosaka K Way D. 2014 Temperature response of photosynthesis in C3, C4, and CAM plants: temperature acclimation and temperature adaptation. Photosynthesis Research 119:101–117. 23801171 10.1007/s11120-013-9874-6

[plw066-B72] Yan W Zhong Y Shangguan Z. 2016 A meta-analysis of leaf gas exchange and water status responses to drought. Scientific Reports 6:20917. 26868055 10.1038/srep20917 PMC4751433

[plw066-B73] Zaehle S Friedlingstein P Friend AD. 2010 Terrestrial nitrogen feedbacks may accelerate future climate change. Geophysical Research Letters 37:L01401.

