# Supplementary material for: Characterizing the drivers of seedling leaf gas exchange responses to warming and altered precipitation: indirect and direct effects
Source: AoB Plants. 2016 Oct 26;8:plw066. doi: 10.1093/aobpla/plw066 (PMC5091920; doi:10.1093/aobpla/plw066)
Supplement: Supplementary Data [file supp_8_plw066_index.html]

Characterizing the drivers of seedling leaf gas exchange responses to warming and altered precipitation: indirect and direct effects — Supplementary Data 

# Characterizing the drivers of seedling leaf gas exchange responses to warming and altered precipitation: indirect and direct effects

## Supplementary Data

files

- Supplementary Data - docx file
