# Supplementary material for: Characterizing the drivers of seedling leaf gas exchange responses to warming and altered precipitation: indirect and direct effects
Source: AoB Plants. 2016 Oct 26;8:plw066. doi: 10.1093/aobpla/plw066 (PMC5091920; doi:10.1093/aobpla/plw066)
Supplement: Supplementary Data [file supp_plw066_aobplants-16064-s02.docx]

**Figure S1. Raw net photosynthesis (*A_n_*) and leaf dark respiration (*R_d_*) data for all species, treatments, and days of year.** Raw net photosynthesis (*A_n_*; µmol CO_2_ m^-2^ s^-1^) and leaf dark respiration (*R_d_*; µmol CO_2_ m^-2^ s^-1^) data for each species during each measurement date in all treatments. Treatments are indicated with different color and symbol combinations. Reduced, ambient, and added precipitation treatments are indicated with brown, grey, and blue points, respectively. Darker colors within a precipitation treatment indicate progressively higher levels of warming. Squares, circles, triangles, and diamonds indicate no, low, medium, and high warming, respectively.
